# Mechanistic insight into the antidiabetic effects of *Ficus hispida* fruits: Inhibition of intestinal glucose absorption and pancreatic beta-cell apoptosis

**DOI:** 10.1371/journal.pone.0337465

**Published:** 2025-12-01

**Authors:** Nusaiba Jahan, Noimul Hasan Siddiquee, Rifayat Ara Khanom Riva, Nur Uddin Chowdhury, Marjanur Rahman Bhuiyan, Khondoker Shahin Ahmed, Hemayet Hossain, Sadikur Rahman Shuvo, AFM. Shahid Ud Daula

**Affiliations:** 1 Department of Pharmacy, Noakhali Science and Technology University, Sonapur, Noakhali, Bangladesh; 2 Department of Microbiology, Noakhali Science and Technology University, Noakhali, Bangladesh; 3 Chemical Research Division, BCSIR Laboratories, Bangladesh Council of Scientific and Industrial Research (BCSIR), Dhaka, Bangladesh; 4 Institute of Food Science and Technology (IFST), Bangladesh Council of Scientific and Industrial Research (BCSIR), Dhaka, Bangladesh; 5 Department of Pharmacy, Jashore University of Science and Technology, Jashore, Bangladesh; Universidade Federal do Para, BRAZIL

## Abstract

The worldwide health impact of Type 2 diabetes mellitus (T2DM) is marked by the dysregulation of glucose metabolism caused by α-glucosidase-mediated carbohydrate degradation and pancreatic β-cell apoptosis through caspase-3 activation. This study aimed to thoroughly investigate the potential roles and mechanisms of *Ficus hispida* fruits methanolic extract (FhME) and its phytoconstituents in combating T2DM by experimental and computational methods. In-vitro investigations demonstrated that, FhME exhibited notable α-glucosidase inhibitory activity compared to acarbose, as indicated by its low IC_50_ value of 850 µg/mL. Furthermore, phytochemical analysis of FhME using the HPLC-DAD technique, combined with a review of previous literature, identified and quantified a total of 26 polyphenolic compounds. The network pharmacological investigation of FhME phytoconstituents identified 70 target genes associated with T2DM, where caspase-3 emerged as a key target. GO enrichment analysis, conducted using SRplot, highlighted key pathways, including apoptosis, lipid and atherosclerosis, and chemical carcinogenesis-receptor activation. Subsequently, molecular docking of caspase-3 with phytochemicals demonstrated strong binding affinity. Post-docking MM-GBSA study identified alpinumisoflavone and chlorogenic acid as exceptionally stable compounds. Molecular dynamics simulations conducted over 200 ns demonstrated that gallic acid and alpinumisoflavone produced the most stable complexes with caspase-3. These findings designate *F. hispida* fruits as a potential natural medicinal agent for Type 2 diabetes treatment, functioning through dual mechanisms of α-glucosidase inhibition and caspase-3 modulation of the apoptotic signaling pathway of the beta cells.

## 1. Introduction

Healthcare professionals are quite anxious about the metabolic disease- Type 2 diabetes mellitus since it is becoming more and more frequent in both developed and underdeveloped countries. Based on a recent survey, 451 million individuals globally (18–99 years old) had Type 2 diabetes mellitus in 2017, and by 2045, that figure is likely to rise to 963 million [1]. Prolonged hyperglycemia in diabetic patients can lead to a variety of potentially fatal consequences, such as coronary artery disease, cardiovascular disease, kidney failure, blindness, neurodegenerative disorders, and reduced life expectancy [2,3].

Till now, Type 2 diabetes mellitus is deemed to be a persistent and slowly advancing disease, that cannot be cured fully with any existing management options- including lifestyle adjustments, synthetic oral hypoglycemic drugs (biguanides, SGLT-2 inhibitors, GLP-1 receptor agonists), along with combination regimens [4,5]. Oral synthetic hypoglycemic medications have been used to treat insulin-resistant type 2 diabetes, but they have undesirable side effects and lose their efficacy with time. Due to the availability of plants, minimal danger of side effects, and, most importantly, low cost of the treatment, controlling hyperglycemia with herbal medicines can be seen as a realistic strategy. Numerous reports suggest that plant extracts have potential hypoglycemic effects through distinct underlying mechanisms, especially in animal models and in-vitro systems [6–9]. Thus, the main goal of this research is to identify more cost-effective and safe alternatives for treating T2DM that may result in fewer side effects and, consequently, improve patient compliance.

*Ficus hispida*, commonly known as the “hairy fig,” is a of fig tree found in many parts of Southeast Asia, including India, Bangladesh, Indonesia, and Malaysia. In traditional medicine, various parts of the *F. hispida* tree have been used to treat a various ailments. [10–12]. For example, the leaves are used to treat skin diseases, such as eczema and psoriasis; the bark is used to treat fever and diarrhoea; the roots are used to treat toothaches and other dental problems; the fruit is used as a laxative and to treat respiratory problems. In addition to its medicinal uses, the fruit is also used in some traditional recipes and is eaten fresh or dried. Phytochemical research has identified terpenoids, alkaloids, flavonoids, phenylpropionic acid, sterols, phenols, and glycosides in *F. hispida* [10,11]. Gosh et al. observed that the ethanolic extract of *F. hispida* bark reduced blood glucose levels and enhanced peripheral glucose absorption [13]. Following therapy with *F. hispida*, the quantity of glycogen in the skeletal muscle, liver, and heart muscle also increased. In alloxan-induced diabetic mice, the methanolic extract of *F. hispida* leaves exhibited a potent antidiabetic action by reducing blood sugar, cholesterol and triglyceride levels [14]. In the earlier study, triterpenoids like betulinic acid; flavonoids like 3′-formyl-5, 7-dihydroxy-4′-methoxyisoflavone, 5,7-dihydroxy-4′-methoxy-3′-(3-methyl-2-hydroxybuten-3-yl)isoflavone, isowigtheone hydrate and alpinumisoflavone; phenolpropionic acids like chlorogenic acid, chlorogenine glycoside, chlorogenic acid methyl ester, and; benzoic acid derivatives like protocatechuic acid and gallic acid; alkaloids including murrayaculatine; steroids like sitosterol 3-O-β-D-glucopyranoside; coumarin such as 7-Hydroxy-6-[2-(R)-hydroxy-3-methyl-but-3-enyl]coumarin were isolated and identified from the *F. hispida* fruits [15]. However, there are no scientific studies on the impact of *F. hispida* fruits on blood glucose levels and how it can ameliorate apoptosis-induced pancreatic β-cell destruction. Consequently, the goal of the current study was to examine the hypoglycemic effects of a methanol extract of *F. hispida* fruits and to evaluate how the phytoconstituents of this plant block the effector genes or proteins associated with the pathogenesis of Type 2 diabetes mellitus. So to further investigate the potential mechanism of antidiabetic action, network pharmacology, molecular docking, and dynamic simulation studies were accomplished between the phytoconstituents and the target proteins (α-glucosidase and caspase-3), which play important roles in the onset of Type 2 diabetes.

## 2. Materials and methods

### 2.1. Sample preparation

Fruits of *F. hispida* were collected from Demra, Dhaka, Bangladesh, in November 2022. Sample specimens of the plants were identified by Sarder Nasir Uddin, the chief scientific officer at the Bangladesh National Herbarium, Mirpur, Dhaka, with an accession ID (DACB Accession No. 49484). Following the collections, the fruits underwent a thorough cleaning to eliminate any unwanted particles. Subsequently, they were left to dry naturally at room temperature, avoiding direct sunlight exposure, for seven days. Finally, the dried fruits were ground into a fine powder. The methanol was used to extract the fine powder. In order to get a pure filtrate, the suspension was passed through a Whatman filter paper. The liquid passed through a filter was subsequently condensed using a rotary evaporator to obtain the unrefined methanolic extract of *F. hispida*. The dark-brown extract was stored at a temperature of 4°C until additional testing could be conducted.

### 2.2. In-vitro antidiabetic study

#### 2.2.1. α-glucosidase inhibitory assay.

α-glucosidase inhibition assay was performed according to the method described by Oboh et al. (2014) with some modifications [16]. At first, 50 μL of potassium phosphate buffer (PBS) with a pH of 6.8 and 10 μL of enzyme solution (1 unit/mL) were combined with 20 μL of either extract (FhME) or a standard (acarbose). The solutions were thereafter placed in an incubator (37°C) for 15 minutes. Subsequently, a solution of p-nitrophenyl-α-D-glucopyranoside (5 mM) in a phosphate buffer (0.1 M) was introduced into a 50 μL portion of the mixture. Following a 5-minute incubation period at a temperature of 25°C, the spectrophotometer was employed to quantify the absorbance at a wavelength of 405 nanometers. Following that, the IC₅₀ values for both the test sample (FH fruit extract) and the standard drug (Acarbose) were calculated from non-linear regression analysis (dose–response inhibition curves) using GraphPad Prism v8.0.2, across the concentration window of 0.01 to 2.5 mg/ml.

### 2.3. HPLC-DAD anaylsis

An analytical procedure which facilitates the segregation and depiction of phytoconstituents of methanolic extract of *F. hispida is carried out*. It incorporates the utilization of Shimadzu LC-20A series HPLC system (Tokyo, Japan) issued with degasser (DGU-20A5), SIL-20A automatic sampler, CTO-20A heating furnace for the column, SPD-20A photo diode array detector, and Luna 5 µm C18 Phenomenex LC column 250 × 4.6 mm as a stationary phase, directed at a fixed 33°C and 270 nm by using an LC solution software system [17,18]. The mobile phase composed of A (1% acetic acid in acetonitrile) and B (1% acetic acid in water) with gradient elution: 0.01−20 min (5−25% A), 20−30 min (25−40% A), 30−35 min (40−60% A), 35−40 min (60−30% A), 40–45 min (30–5% A), and 45–50 min (5% A) was used in this study. The phenolic compounds present in the methanolic extract of FH were identified by evaluating the resulting peak regions and UV spectrum using the reference standard as a guide. In order to show the concentration of each metabolite by the peaks, this process produced a result in mg/100 g of dry extract.

### 2.4. Network pharmacology

The focal point of Network Pharmacology lies in depicting both the pharmacological targets and the disease phenotype targets, figuring out the mechanism of drug-disease associations, and examining the network to identify the channels through which network targets and system regulation are connected [19].

#### 2.4..1. Potential genes of phytoconstituents.

Several gene datasets of each phytoconstituent obtained from *Ficus hispida* fruits were retrieved in TSV format using a free online server: STITCH v5.0 (http://stitch.embl.de/) and SwissTargetPrediction Bioinformatics Platform (http://www.swisstargetprediction.ch/) while the search was limited to *Homo sapiens*. Then, compound genes having probability score of ≥0.4 were screened out to continue with further proceedings.

#### 2.4.2. Genes related to type 2 diabetes mellitus.

The genes concerned with Type 2 diabetes mellitus were obtained by employing two online database: GeneCards v5.20 (https://www.genecards.org/) and Online Mendelian Inheritance in Man- OMIM (https://www.omim.org/). These databases were then processed to cancel out the duplicate genes at the moment of compilation of genes.

#### 2.4.3. Common genes analysis.

The obtained genes from the above procedures were denoted as two different datasets and subjected to Bio-informatics & Evolutionary Genomics (https://bioinformatics.psb.ugent.be/webtools/Venn/), a free online tool to bring about the common target genes having dual involvement in between the secondary metabolites and T2DM.

#### 2.4.4. Network construction.

After analyzing and assembling the prevalent target genes between each phytoconstituent and common genes by Bio-informatics & Evolutionary Genomics and Microsoft Excel, respectively, the TSV file of this database was imported to Cytoscape v3.10.2, where phytoconstituents and target genes were elucidated as source and target nodes independently. Each edge represents the interaction between a phytoconstituent and its target genes. Target genes were defined in round nodes, while the diamond nodes constitute the phytoconstituents. By implementing the degree attribute, the size and colour of each node were elucidated according to the lower to higher significance [19]. This network provides a clear estimation of densely connected target nodes associated with the pathogenesis of T2DM.

#### 2.4.5. GO enrichment.

Following the acquisition of a set of key target genes associated with T2DM, these were brought along with other necessary data into the input frame of SRplot (https://www.bioinformatics.com.cn/srplot) free website to appraise the top 10 signalling pathways associated with T2DM and to evaluate the number of genes involved in each specific pathway. By adjusting and formatting the default parameters, the appearance of the resultant graph was transposed [20]. Consequently, a bubble plot with an Enrichment Score on the x-axis and the names of pathways on the y-axis was created.

### 2.5. Molecular docking

#### 2.5.1. Molecular docking with α-glucosidase.

The crystalline structure of experimentally resolved human α-glucosidase (PDB ID: 5NN8) was acquired from the RCSB PDB database in order to conduct molecular docking. Initially, the protein was processed using PyMol to remove molecules of water and other unwanted hetero atoms. Energy minimization of protein structure was performed using the Swiss PDB viewer (version 4.1.0). The protein’s PDB file underwent modifications through the Autodock program, which included polar hydrogens and Kollman charges [21]. The grid box having a centre X: −7.893, Y: −29.522, and Z: 92.792 and a dimension of 40 at the X, Y, and Z axes with an exhaustiveness of 8 was generated at the active site of the protein. Subsequently, the protein structure was saved in PDBQT format for the purpose of conducting docking studies. From the PubChem database, the structures of polyphenols (ligands) were downloaded in SDF format. At the very end, docking was carried out using Autodock Vina (v.1.2.0).

#### 2.5.2. Molecular docking with caspase-3.

The 3D crystallized formation of caspase-3 affixed with isoquinoline-1,3,4-trione derivative inhibitors was retained from the RCSB Protein Data Bank (https://www.rcsb.org/), having four chains (chains A, B, C, and D) with PDB ID: 3DEI in PDB format. The X-ray diffraction procedure, with a resolution of 2.80 Å, was utilized to ascertain both the molecular and atomic structure of the crystallized protein [22]. By using Maestro v11.4, water molecules, side chains, and hetero atom molecules were removed, which were subsequently processed through Schrödinger Protein Preparation Wizard 2020−3 to optimize the protein structure.

After network pharmacology analysis, four ligands having interactions with caspase-3 were selected and therefore loaded in SDF format using PubChem (https://pubchem.ncbi.nlm.nih.gov). The LigPrep function of Maestro v11.4 was employed to prepare the ligands. As a consequence, to modify the ionization state of ligands, pH 7.0 in conjunction with Epik version 5.3 was used [23]. In the preparation of proteins and ligands, the OPLS-3e force field was implemented [24].

Applying the OPLS-3e force field in standard precision mode, Schrödinger-Desmond software docked 04 ligands with the target macromolecule using GLIDE v8.8 and Maestro v11.4 [24,25]. Based on residues observed at binding locations, the receptor grid was used to define the box range X = −46.387, Y = 15.352, and Z = −22.133. Target protein and ligand binding energies were measured, and residues and chemical bonds involved in ligand binding were displayed using the Maestro viewer [26].

### 2.6. ADMET analysis

Pharmacokinetic studies are commonly used to detect and exclude inappropriate compounds while collecting critical data on medication absorption, distribution, metabolism, and excretion in order to determine the connection between dosage, dosage form, and systemic exposure [27,28]. According to Lipinski’s rule of five, ADME attributes show the accessibility of compounds throughout the body [29]. Predicting compound toxicity can enhance the optimization of lead compounds and reduce the likelihood of failure in drug development [27,30]. The free web tools SwissADME (http://www.swissaT1DMe.ch) and PkCSM (http://structure.bioc.cam.ac.uk/pkcsm) were used for in-silico ADME screening and toxicity properties evaluation. SwissADME was utilized to predict pharmacokinetic properties, including lipophilicity, solubility, GI absorption, and BBB penetration, while pkCSM was employed to assess both ADME and toxicity parameters, such as AMES toxicity and hepatotoxicity, with toxic compounds excluded from the study.

### 2.7. Post-docking MM-GBSA

Molecular mechanics generalized born surface area (MM-GBSA), being one of the fastest force-field based methods, was used to perform re-scoring of binding free energy of selected four compounds each in conjunction with the protein due to the lack of accuracy and precision of scores obtained during molecular docking procedure [31,32]. After fixing the grid box to the previous position, dG Bind, dG Bind Coulomb. dG Bind Covalent, dG Bind Hbond, dG Bind Lipo, dG Bind Packing, dG Bind SelfCont, dG Bind Solv GB, dG Bind vdW etc. were computed by employing Prime MM-GBSA module of the Schrödinger Maestro v11.4 and Glide v8.8 with OPLS-3e as force field of the top scoring compounds in docking [24].

### 2.8. Molecular dynamics simulation

Molecular dynamics for a period of 200 ns was carried out to determine the physical integrity of the top selected ligands within the active site of enzyme-ligand complex at a chosen physiological environment [33]. After pre-processing the protein with Schrödinger protein preparation wizard 2020−3, the complex was processed through the Desmond module of Schrödinger suit. During this process, the system was subjected to an orthorhombic box with a span of (10 × 10 × 10 **Å**^**3**^) to incorporate the system within the Simple Point Charge (SPC) water model [34]. A salt concentration of 0.15M with Na^+^ and Cl^-^ ions was incorporated to counteract the system. The simulation was consummated under an NPT ensemble at 300 K and 1.01325 bar pressure while OPLS-3e as force field was implemented [35]. For each complex, we first allowed the system to relax, and then we recorded the final production at 200-ps intervals while applying an energy of 1.2. From the 200 ns MD trajectory, we analyzed protein root mean square deviation (Protein RMSD), ligand RMSD, protein root mean square fluctuation (RMSF), radius of gyration (Rg), solvent accessible surface area (SASA), polar surface area (PolSA), and protein-ligand interaction using a simulation interaction diagram.

## 3. Results

### 3.1. Antidiabetic activity of *F. hispida* fruit extract

#### 3.1.1. In vitro α-glucosidase inhibition assay.

**Fig 1:**
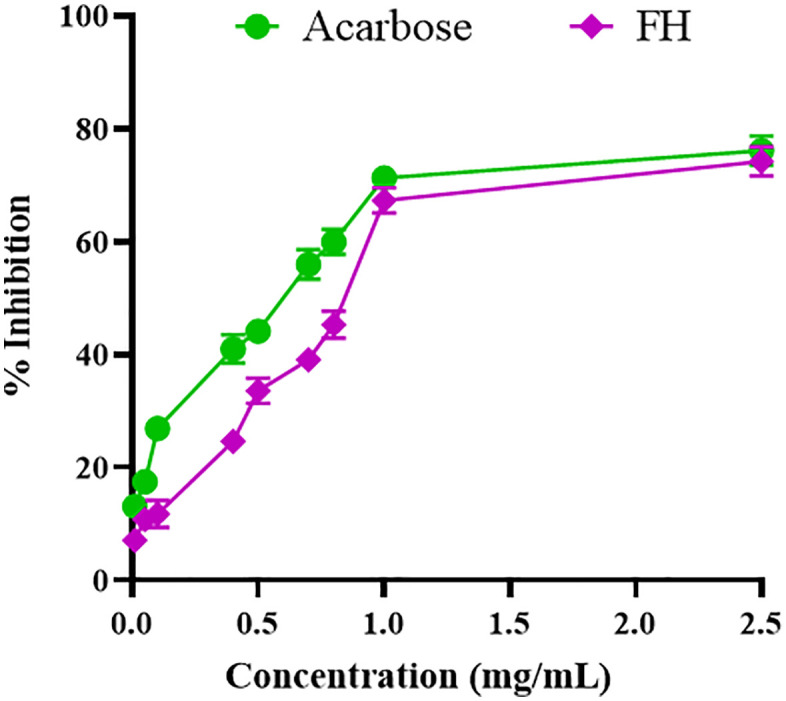
Effect of *F. hispida* methanol extract on α-glucosidase.

The inhibitory activity of FhME against α-glucosidase is shown in **Fig 1 and** **S1 Table**. The suppressive impact of the FhME was compared to the conventional α-glucosidase inhibitor acarbose. Both doses of FhME and acarbose exhibited a dose-dependent response, whereby the magnitude of the effect increased with higher concentrations. In contrast to the standard, the fruit extract demonstrated a potent inhibitory effect on the enzyme. The IC_50_ values for acarbose and fruit extract were 0.59 mg/mL and 0.85 mg/mL, respectively.

### 3.2. Phytochemical analysis

#### 3.2.1. Phenolic composition.

**Fig 2:**
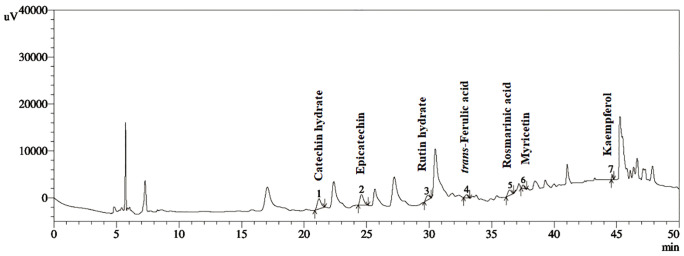
HPLC-DAD chromatogram for *F. hispidia* fruit extract.

**Fig 2** illustrates the phenolic profile of FhME. Through the comparison of the extract’s retention duration with 16 standard stock solutions, a total of seven polyphenolic components were identified in the fruit extract. The phenolic component most abundantly found in the fruit extracts was catechin hydrate (25.32 mg/100 g). (-) Epicatechin (18.91 mg/100 g), rosmarinic acid (4.87 mg/100 g), rutin hydrate (3.82 mg/100 g), and trans-ferulic acid (1.77 mg/100 g) were also discovered in the fruit extract in appreciable quantities. On the other hand, myricetin (1.24 mg/100 g) and kaempferol (0.48 mg/100 g) were found to be present in the least amount (**S2 Table**).

### 3.3. Network pharmacology analysis

#### 3.3.1. Mining of phytoconstituents targets.

A cumulative dataset of total 209 compound genes with repetition of genes were extracted from 13 phytoconstituents by using two different databases: STITCH v5.0 and SwissTargetPrediction. These were further sorted out in compliance with the combined score to reveal that 83 target genes have a probability of being potential targets.

#### 3.3.2. Screening of disease genes.

From OMIM database, a set of genes of T2DM were acquired which were supplemented by genes obtained through GeneCards. After eliminating duplication, this created another dataset of 6620 genes having involvement in the pathogenesis of T2DM.

#### 3.3.3. Venn diagram.

**Fig 3:**
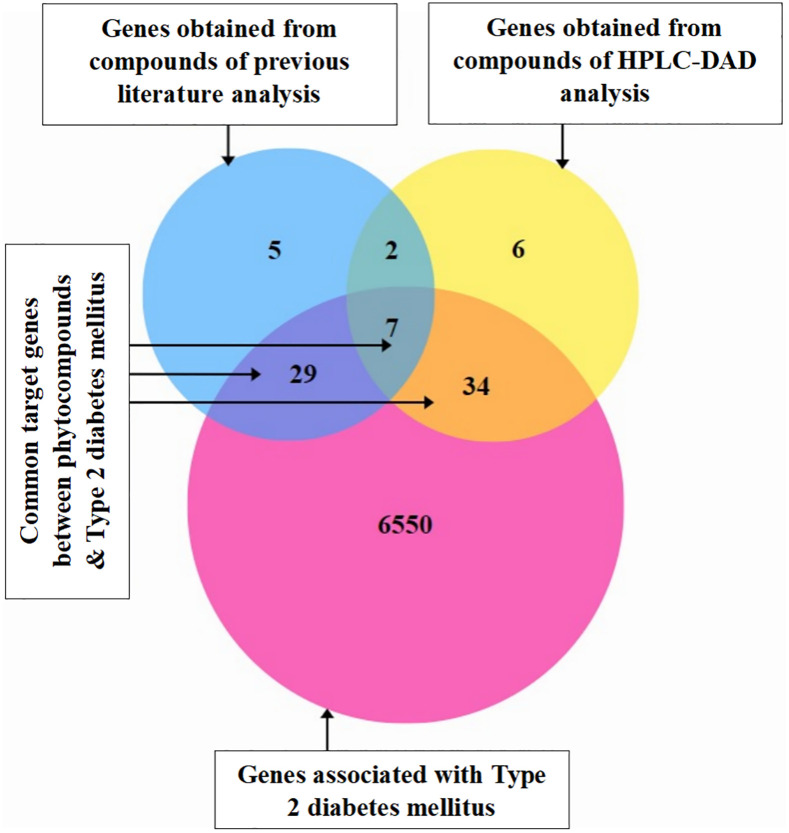
Common target genes between phytoconstituents and disease genes.

From **Fig 3** it was disclosed that about 70 genes have mutual participation between the two previously mentioned datasets. Among the phytoconstituents genes, 43 were extracted from compound analysis by previous literature, while 49 genes were assembled from compounds analyzed through HPLC-DAD technique.

#### 3.3.4. Network construction.

**Fig 4:**
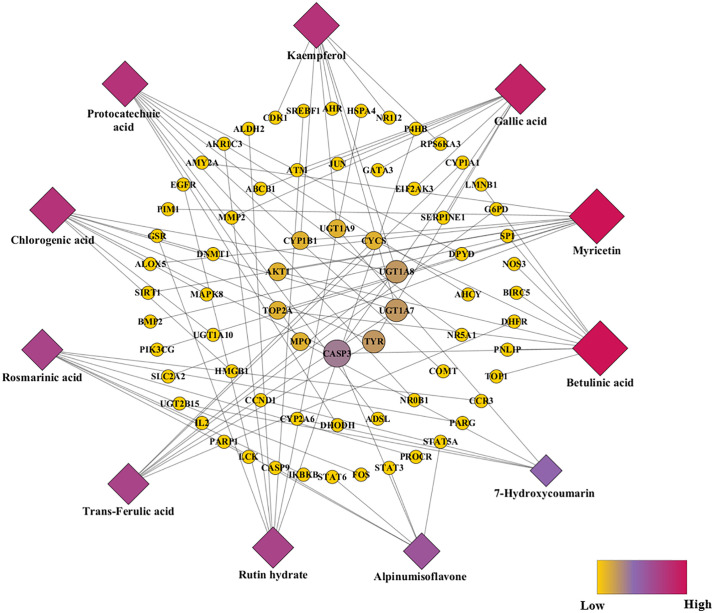
Network interaction between phytoconstituents and target genes.

In **Fig 4**, a network illustrating phytoconstituents of *Ficus hispida* along with its target genes and disease genes associated with T2DM to interpret the potential of FH as an anti-diabetic was contrived. Each of the 11 phytoconstituents was found to interact with more than one disease gene. Gallic acid, myricetin and betulinic acid exhibited the highest number of connectivity while alpinumisoflavone and 7-hydroxycoumarin had the least interaction (**S5 Table**). Among the disease genes, CASP3, TYR, UGT1A7, and UGT1A8 were depicted to be the highly engaged genes with several phytoconstituents. CASP3 was selected for further experiment in docking study, which has the highest score of “edge count” in interaction with betulinic acid, gallic acid, chlorogenic acid, and alpinumisoflavone.

#### 3.3.5. GO enrichment analysis.

**Fig 5:**
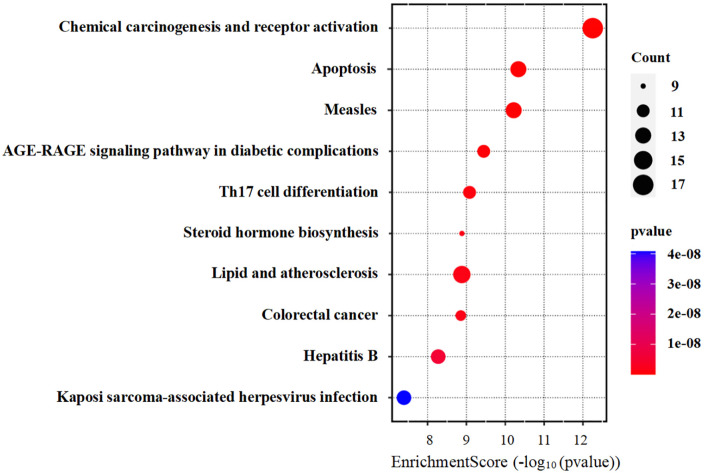
GO enrichment bubble plot.

From **Fig 5**, this bubble plot showed which pathways were most relevant in the pathogenesis of T2DM along with their respective number of genes. Pathways having >12 genes were Apoptosis, Measles, Lipid and atherosclerosis, and Chemical carcinogenesis-receptor activation. On the contrary, pathways with <12 genes were Th17 cell differentiation, AGE-RAGE signaling pathway in diabetic complications, Steroid hormone biosynthesis, Colorectal cancer, Hepatitis B, and Kaposi sarcoma-associated herpesvirus infection. Apoptosis is one of the leading contributing factors in the pathogenesis of Type 2 diabetes mellitus.

### 3.4. Molecular docking

#### 3.4.1. α-glucosidase and ligand docking assessment.

Docking experiments were conducted to predict the nature and intensity of the interaction between the α-glucosidase enzyme and the phytochemicals discovered by HPLC-DAD (**S2 Table**) as well as the chemical constituents previously isolated (**S4 Table**) by Zhang et al. (2018) and Cheng et al. (2021) from *F. hispida* fruits [15,36]. Outputs for all ligands and standards are indicated by binding energy values (kcal/mol) and represented in **Table 1**. Out of 15 docked compounds, 09 phytochemicals showed higher binding affinity (−7.1 to −6.7 kcal/mol) than the standard acarbose (−6.6 kcal/mol). Among the previously identified compounds, chlorogenic acid, chlorogenic acid methyl ester, (6s,9r)-roseoside, alpinumisoflavone, isowigtheone hydrate also demonstrated higher binding scores than the acarbose.

**Table 1:** Docking score of identified phytochemical compounds and standard drug (acarbose) in the binding site of α-glucosidase (5NN8) computed via Autodock vina software.

Chemical Compound Name with CID	Binding Energy (kcal/mol)
(-) Epicatechin (CID: 9064)	−7.1
Chlorogenic acid (CID: 1794427)	−7.1
Chlorogenic acid methyl ester (CID: 6476139)	−7.1
(6S,9R)-Roseoside (CID: 9930064)	−6.9
Alpinumisoflavone (CID: 5490139)	−6.8
Catechin hydrate (CID: 107957)	−6.8
Rosmarinic acid (CID: 5281792)	−6.8
Isowigtheone hydrate (CID: 66728267)	−6.7
Myricetin (CID: 5281672)	−6.7
Acarbose (CID: 41774)	−6.6
Kaempferol (CID: 5280863)	−6.4
Rutin hydrate (CID: 16218542)	−6
Trans-Ferulic acid (CID: 445858)	−5.7
Betulinic acid (CID: 64971)	−5.5
Gallic acid (CID: 370)	−5.1

#### 3.4.2. Caspase-3 and ligand docking assessment.

Four drug-like compounds from *F. hispida* extract, selected using network pharmacology, were subjected to molecular docking studies. The three compounds that performed the best in terms of docking score, gallic acid (CID: 370), chlorogenic acid (CID: 1794427), and alpinumisoflavone (CID: 5490139) have been selected with energy values of −4.706, −4.42, and −3.745 kcal/mol, respectively (**Table 2**).

**Table 2:** List of compound name and binding energy (kcal/mol) of the selected top four selected ligands with caspase-3.

Chemical Compound Name with CID	Binding Energy (kcal/mol)
Gallic acid (CID: 370)	−4.706
Alpinumisoflavone (CID: 5490139)	−4.42
Chlorogenic Acid (CID: 1794427)	−3.745
Betulinic acid (CID: 64971)	0.191

**Fig 6:**
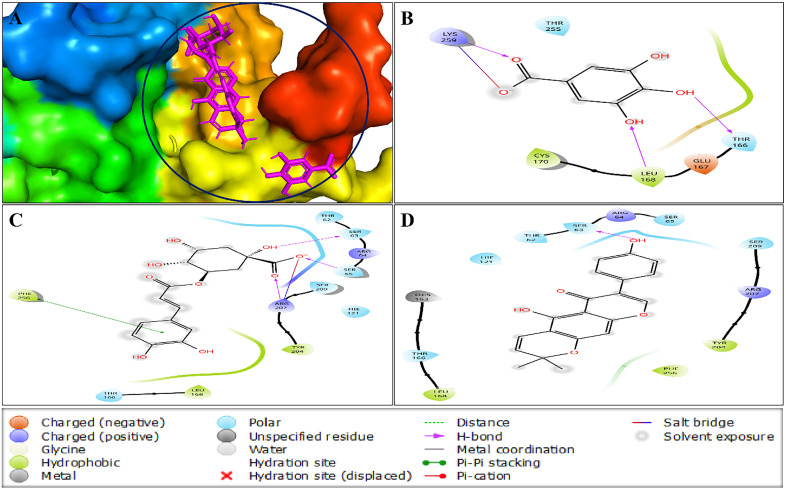
Interaction between caspase-3 enzyme and best three docking score compounds, represented in 3D (A) and 2D (B,C,D) format where the compounds, B, C, and D represent gallic acid (CIDs: 307), chlorogenic Acid (CID:1794427) and alpinumisoflavone (CID: 5490139), respectively.

The top docking scores of the three compounds chosen were taken out for additional examination. Molecular interactions were visualized using the Schrödinger suite’s Maestro module, which helped to identify polar, hydrophobic, hydrogen, and electrostatic bonds between receptors and ligands. The selected compounds coordinated with the shared amino acid residues of the protein during molecular docking. The common amino acids involved in H-bonding, polar bonding, and hydrophobic interactions are SER63, THR166, and LEU168, respectively, with each of the three compounds interacting uniquely with a specific amino acid, as illustrated in **Fig 6** and detailed in **S6 Table**.

### 3.5. Evaluation of pharmacokinetics and toxicities characteristics

The good pharmacokinetic features of all the drugs under consideration indicate a low likelihood of failure in clinical trials. The toxicity and side effects of medications are among the primary issues that lead to their failure during development. In this study, three compounds – gallic acid, chlorogenic acid, alpinumisoflavone with CIDs of 307, 1794427, and 5490139 passed Lipinski’s rule of five and showed favorable pharmacokinetics and toxicity properties, indicating a low probability of failure in a clinical trial presented in **S7** and **S8 Tables**.

### 3.6. Post-docking MM-GBSA analysis

**Fig 7:**
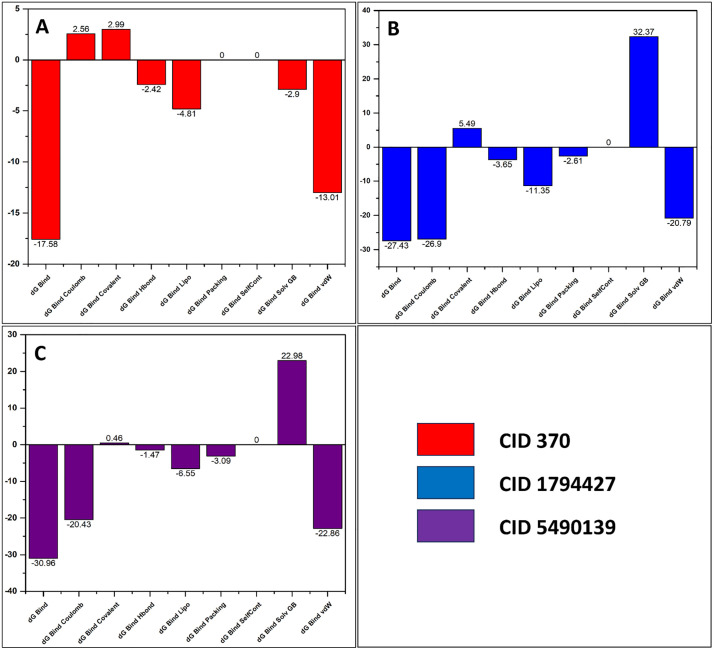
Post-docking MM/GBSA of selected three compounds. Gallic acid (CID: 370), chlorogenic acid (CID: 1794427), alpinumisoflavone (CID: 5490139).

The protein-ligand conjugates obtained from the docking experiment were subjected to study MM-GBSA to re-score the complexes in accordance with binding free energy. The lower the binding free energy, the stronger the interaction between the ligand and protein exhibits [37]. The resultant values of post-docking MM-GBSA were depicted in **S9 Table**. dG binding energy, being the most prominent parameter states that, gallic acid (CID: 370), chlorogenic acid (CID: 1794427), and alpinumisoflavone (CID: 5490139) have varying values which range from −30.96 to −17.58 kcal/mol. Among these ligands, alpinumisoflavone (−30.96 kcal/mol) shows the highest binding affinity with caspase-3, indicating it can interact with the target protein for a long time (**Fig 7**). But the compound CID: 1794427 also showed a negative binding energy of −27.43 kcal/mol, which indicates an almost similar result to CID: 5490139.

### 3.7. Molecular dynamics analysis

To establish structural integrity and intermolecular affinity of protein-ligand complex, a simulation in a manipulated environment for 200 ns was conducted.

#### 3.7.1. Protein RMSD.

**Fig 8:**
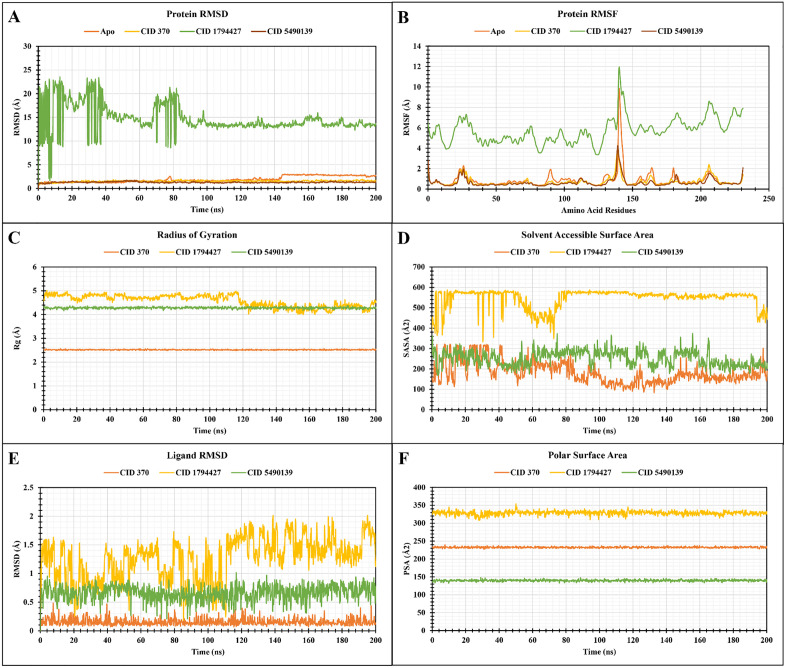
Graph representing A) RMSD B) RMSF C) Rg D) SASA E) Ligand-RMSD F) PSA values of apo-protein & compound complexes based on 200 ns simulation, where compounds include- gallic acid (CID: 370), chlorogenic acid (CID: 1794427), alpinumisoflavone (CID: 5490139).

By computing the average value change caused by the displacement of atoms from a definite frame in contrast to a reference frame, the stability and constancy of conformation can be investigated [26,38]. From RMSD graph analysis, the mean RMSD values of apo-protein, gallic acid, chlorogenic acid and alpinumisoflavone were 1.89 Å, 1.52 Å, 14.75 Å, and 1.30 Å, respectively. The lowest RMSD values for gallic acid, chlorogenic acid and alpinumisoflavone are 0.721 Å, 1.984 Å, and 0.869 Å at frame numbers 2, 34, and 19, respectively and the highest scores were 1.92 Å, 23.542 Å, and 1.735 Å at 961, 64, and 262 frames, respectively. The allowable RMSD value should lie below 3 Å to have a desirable conformation of the complex [39]. From **Fig 8A**, it is seen that except chlorogenic acid, the remaining two ligands when complexed with apo-protein showed acceptable values. Moreover, while the native structure of apo-protein encountered slight variation during 75–80 ns and 143–200 ns, the complexes formed with the protein with gallic acid and alpinumisoflavone resulted in a more stable complex.

#### 3.7.2. Protein RMSF.

RMSF value characterizes the oscillation in the regional area of the atoms that are present in each amino acid residue to compute the flexibility of the complex [33]. The average RMSF values for apo-protein, gallic acid, chlorogenic acid, and alpinumisoflavone were 0.893 Å, 0.733 Å, 5.73 Å, and 0.66 Å, correspondingly. The lowest RMSF values of gallic acid, chlorogenic acid, and alpinumisoflavone with their respective residual amino acid are 0.339 Å (VAL 117), 3.369 Å (PHE 158) and 0.312 Å (LEU 119) while the highest RMSF values are 3.89 Å (GLU 173), 11.81 Å (LYS 186), and 4.228 Å (GLU 173). In **Fig 8B**, the highest peaks for all three ligands and apo-protein were observed between 135–145 residues. Both gallic acid and alpinumisoflavone exhibited notable spikes at the same amino acid residue which also specified a more rigid and stable conformation than chlorogenic acid.

#### 3.7.3. Radius of gyration (Rg).

Rg, a benchmark of protein compactness, provides an estimation of how tightly the ligand forms a bond at the active cavity of a given protein during simulation [38,40]. The average value of Rg of gallic acid, chlorogenic acid and alpinumisoflavone followed- 2.52 Å, 4.58 Å, and 4.28 Å in order. The ranges of Rg value for gallic acid, chlorogenic acid, and alpinumisoflavone were 2.48 Å to 2.565 Å, 4.001 Å to 5.004 Å, and 4.16 Å to 4.374 Å, respectively. From **Fig 8C**, it is depicted that, among the three ligands, both gallic acid and alpinumisoflavone demonstrated undeviating mobility throughout the process while chlorogenic acid did not confine to consistency having diversified fluctuation from 115 to 125 ns.

#### 3.7.4. Ligand RMSD.

The mean ligand RMSD values were 0.153 Å, 1.26 Å, and 0.66 Å for gallic acid, chlorogenic acid, and alpinumisoflavone, correspondingly. The lowest values for gallic acid, chlorogenic acid, and alpinumisoflavone were found at 244, 427, and 377 frame numbers having 0.07 Å, 0.197 Å, and 0.208 Å values, respectively. On the contrary, the highest values were 0.485 Å, 2.012 Å, and 1.01 Å at 38, 976, and 584 frame numbers. From **Fig 8E**, all the ligands exhibited favorable RMSD values while simulated in their inherent form. The results showed that alpinumisoflavone and gallic acid were preferable to chlorogenic acid, which fluctuated widely.

#### 3.7.5. SASA and PolSA.

SASA quantifies the accessibility of the surface of the ligand-protein complex to the water solvent by analyzing the conformational changes occurring during the interaction [33]. The SASA values on average showed 181.09 Å^2^, 545.93 Å^2^, and 252.92 Å^2^ for gallic acid, chlorogenic acid, and alpinumisoflavone, independently. While the lowest and highest SASA values were 84.167 Å^2^ and 320.964 Å^2^ at 663 and 38 frame numbers for gallic acid, similar to chlorogenic acid were 335.795 Å^2^ and 586.818 Å^2^ at 152 and 471 number frames and for alpinumisoflavone the values were 168.233 Å^2^ and 374.65 Å^2^ at 831 and 778 frame numbers. The average SASA value of the complex system fell between 180 Å^2^ and 550 Å^2^. In **Fig 8D**, the compounds having favourable SASA values were gallic acid and alpinumisoflavone.

PolSA represents the cumulative surface area occupied by nitrogen and oxygen atoms in their bound state with hydrogen [41]. As per the calculation observed in **Fig 8F**, the PolSA values ranged from 227.473 Å^2^ to 239.531 Å^2^ for gallic acid, 308.27 Å^2^ to 353.928 Å^2^ for chlorogenic acid, and 132.439 Å^2^ to 148.023 Å^2^ for alpinumisoflavone. The average PolSA values according to this order were 232.59 Å^2^, 328.47 Å^2^, and 139.90 Å^2^. The most accepted compound with the lowest PolSA value was alpinumisoflavone (**Fig 8F**).

#### 3.7.6. Protein-ligand contact analysis.

**Fig 9:**
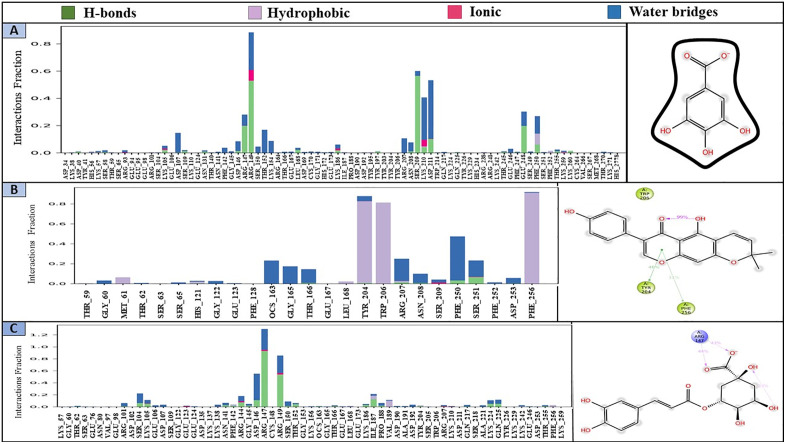
The protein-ligand interaction produced during 200 ns simulation represented by a stacked bar chart with their respective 2D image. **A)** gallic acid (CID: 370), **B)** alpinumisoflavone (CID: 5490139) & **C)** chlorogenic acid (CID: 1794427).

The Simulation Interaction Diagram (SID) is used to inspect a handful of bonds formed between the selected ligands and amino acid residues by dint of H-bonds, hydrophobic, ionic, and water bridges [42,43]. The H-bond, being the noteworthy interaction, guides drug absorption, metabolism and specificity [42]. **Fig 9** represents if there is any interaction present and type of bonds between protein and selected ligands throughout 200 ns simulation. Gallic acid (CID: 370) developed multiple interactions at LYS57 (0.02), LYS105 (0.06), ARG147 (0.28), ARG149 (0.9), LYS186 (0.075), LYS210 (0.4), ASP211 (0.53), PHE250 (0.24), and LYS259 (0.025) residues according to simulation time. Alpinumisoflavone (CID: 5490139) formed more than 2 bonds at TYR204 (0.9), ARG207 (0.25), SER209 (0.03), and SER251 (0.23) residues with their respective interaction fraction (IF). During the simulation, chlorogenic acid (CID: 1794427) created few interactions with protein at residues LYS105 (0.11), GLU123 (0.02), LYS138 (0.04), PHE142 (0.04), ARG144 (0.18), ARG147 (1.3), ARG149 (0.84), THR166 (0.03), ILE187 (0.21), TYR204 (0.025), and ARG207 (0.03). There were some residues which were shared among the three compounds including LYS105, ARG147, ARG149, TYR204, and ARG207. The 2D images of these protein-ligand contacts were illustrated next to the histogram (**Fig 9**). Throughout the simulation, gallic acid emerged as the most stable compound rather than chlorogenic acid and alpinumisoflavone.

## 4. Discussion

Medicinal herbs, their extracts, or individual phytochemicals have been utilized to regulate blood glucose levels since the dawn of time [44]. As Type 2 diabetes has become the world’s most serious health problem, this study focuses on plant-based research and finally assesses the antidiabetic activity of *F. hispida* fruits. Plant and herb extracts that are rich in phenolic compounds have been found to have a diverse spectrum of pharmacological effects such as anti-inflammatory, bactericidal, anti-osteoporosis, antineoplastic, hypoglycemic, antioxidant, and antihelmintic properties [45,46]. Based on both current and previous investigations [15,36], it has been found that *F. hispida* fruit extracts contain a significant quantity of various polyphenolic compounds (**S1** **and** **S2 Tables**). Hence, the antidiabetic effect of the FhME could be attributed to the existence of the aforementioned bioactive components.

An earlier study reported that rutin hydrate and epicatechin exhibit a potent antidiabetic activity [47]. Rosmarinic acid has a significant effect on regulating plasma glucose levels and improving insulin sensitivity in hyperglycemia, as demonstrated by Ngo et al., 2018. [48]. Epicatechin, rutin hydrate and rosmarinic acid were found in considerable amounts in FhME, which could also be responsible for the potent hypoglycemic effects of plant extract.

Chlorogenic acid (CGA) is a natural phenolic compound isolated from the fruits of *F. hispidia* by Zhang et al. [15]. CGA has been studied for its potential health benefits, including its anti-diabetic activity. A previous study reported that CGA supplementation significantly reduced blood glucose levels in diabetic rats [49]. Another study found that CGA reduced insulin resistance and improved glucose tolerance in diabetic mice [50].

In accordance with the presence of multiple signaling networks related to disease, network pharmacology is designed to create a framework which authenticates the interaction between multiple disease genes and drug target genes. This approach unfolds the development of innovative drugs [51]. In this study, network pharmacology revealed several disease genes associated with Type 2 diabetes mellitus and their association with phytoconstituent genes. Among those, CASP3, TYR, UGT1A7, and UGT1A8 were highly associated with most of the phytoconstituent genes. Targeting caspase-3 (CASP-3) has the most prominent role, as it causes gradual loss of pancreatic β-cell, indicating Type 2 diabetes mellitus.

Structure-based molecular docking enables the rapid analysis of a large number of chemical candidates and the classification of those candidates as potential hits capable of interacting with a specific target protein for the management and treatment of a particular illness [52]. Inhibition of both α-glucosidase and caspase-3 remain a powerful strategy in the development of new antidiabetic agents [53]. By preventing the action of these enzymes, degenerative health problems, such as Type 2 diabetes mellitus, can be controlled. Due to this, less glucose is absorbed in the intestines, the release of glucose into the blood is delayed, and the apoptotic cell death of pancreatic β-cell can be diminished [54,55]. Numerous earlier studies suggested that natural phenolic substances extracted from many natural plants are able to block these enzymes [56–58]. Therefore, to examine the antidiabetic mechanism of action, molecular docking was performed between the *F. hispida* phytochemicals and α-glucosidase & caspase-3. Molecular docking of *F. hispida* compounds with α-glucosidase showed that most of the phytochemicals exhibited good binding affinity. The strongest binding affinities for α-glucosidase were found for (-)epicatechin, chlorogenic acid, chlorogenic acid methyl ester, (6s,9r)-roseoside, alpinumisoflavone, rosmarinic acid, catechin hydrate, isowigtheone hydrate, and myricetin having docking score from −7.1 to −6.7 kcal/mol. These findings further suggest that the compounds identified in fruit extract may lessen the release and small intestinal absorption of glucose, hence lowering blood glucose levels, by reducing the breakdown of disaccharides and oligosaccharides. Further docking with caspase-3 found gallic acid (−4.706 kcal/mol), chlorogenic acid (−3.745 kcal/mol), and alpinumisoflavone (−4.42 kcal/mol) having the strongest binding affinity. This study validates the ability of these three compounds to block the effect of apoptosis-induced pancreatic β-cell destruction. The aforementioned compounds therefore possess strong hypoglycemic drug characteristics.

Molecular dynamics (MD) simulations are employed to investigate the structural alterations of a protein when it undergoes ligand binding inside docking complexes. The utilization of MD simulation is a highly effective technique in the field of phytochemical pharmacology, as it offers valuable insights into the intricate interactions between natural compounds and biological systems. This leads to a more comprehensive exploration of binding poses and more precise estimations of affinity, ultimately enhancing the accuracy of the obtained structural data. The lower values of protein RMSD, ligand RMSD, protein RMSF, Rg, SASA, and PolSA scores indicate a larger degree of compactness in the system. The average values of protein RMSD (1.52 Å and 1.30 Å), protein RMSF (0.733 Å and 0.66 Å), Rg (2.52 Å and 4.28 Å) ligand RMSD (0.153 Å and 0.66 Å), SASA (181.09 Å^2^ and 252.92 Å^2^), PolSA (232.59 Å^2^ and 139.90 Å^2^) have recognized gallic acid and alpinumisoflavone respectively, having the least conformational changes, compact binding and stability of apo-enzyme in the presence of ligand-protein complexes that verified the docking studies while, protein-ligand contact analysis explored gallic acid having favorable interaction with multiple amino-acid residues in preference over chlorogenic acid and alpinumisoflavone.

**Fig 10:**
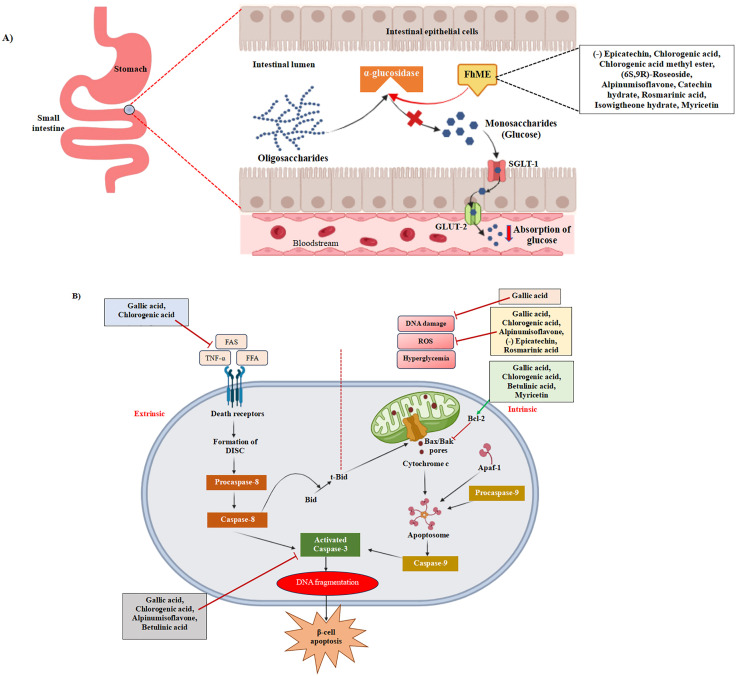
Dual mechanisms of antidiabetic activity of *Ficus hispida* fruits extract through α-glucosidase inhibition and anti-apoptotic activity. **A)** Role of α-glucosidase in breakdown and absorption of glucose where *Ficus hispida* fruits extract acts as α-glucosidase competitive inhibitor. **B)** Role of caspase-3 in pancreatic β-cell destruction and how various phytoconstituents obtained from *Ficus hispida* fruits extract block the apoptotic signaling pathway. Green arrow indicates stimulatory effect, and red arrow indicates inhibitory effects.

As illustrated in **Fig 10A**, α-glucosidase is one of the principal enzymes in the digestion and absorption of carbohydrates (polysaccharides). These polysaccharides are partially cleaved in the mouth by salivary α-amylase enzyme, which are further hydrolyzed to monomer or glucose in the brush border of the small intestinal lumen by α-glucosidase enzyme. Subsequnetly, glucose is translocated to intestinal epithelial cells by sodium/glucose cotransporter-1 (SGLT-1) and, therefore, infiltrates the bloodstream via glucose transporter-2 (GLUT-2) [59]. Thus, blood glucose level is elevated after taking a meal. To date, various synthetic anti-diabetic medications have been established, but long-term application brings about long-term complications including- vomiting, diarrhea, stomach cramps, flatulence, and allergic reaction etc. [60]. This has led to the discovery of traditional medicines that have a strong affinity against α-glucosidase enzyme. In **Fig 10A**, it is stated that, phenolic compounds obtained from *Ficus hispida* fruit extract can act as inhibitors of α-glucosidase, which binds competitively to the active site of the enzyme and downturns the digestion and absorption of glucose in the blood [60].

Apoptosis is the most prominent pathway in the destruction of pancreatic β-cell. Caspase-3 is the effector caspase, which plays a dominant role in the process of apoptosis. Apoptosis consists of two signaling pathways: the extrinsic or cell death receptor pathway and the intrinsic or mitochondrial pathway [55]. The extrinsic pathway is instigated by the binding of circulating external ligands (TNF-α, FFA, FAS, etc.) to the cell death receptors, which trigger the formation of death-inducing signalling complex (DISC) [61]. DISC thus leads to the actuation of caspase-8, which subsequently cleaves and activates caspase-3, leading to DNA fragmentation and apoptotic cell death [61,62]. Caspase-8, on the other hand, cleaves Bid to form a truncated Bid (t-Bid), which prompts the formation of Bax/Bak pores on the mitochondrial surface [63]. This occurrence, in combination with mitochondrial damage by UV, DNA damage, ROS formation, and hyperglycemic events gives rise to an intrinsic apoptotic pathway [62]. Through the Bax/Bak pores, pro-apoptotic proteins, including cytochrome c, are released into the cytoplasm, which gets conjugated with apoptotic protease activating factor-1 (Apaf-1) and procaspase-9 to generate apoptosome [62,63]. This complex activates caspase-9 and, in turn, cleaves and activates caspase-3 to promote cell death [63]. Upregulation of an anti-apoptotic protein, Bcl-2, blocks the formation of Bax/Bak pore [61]. In **Fig 10B**, it is seen that chlorogenic acid, gallic acid, betulinic acid, and myricetin, found almost exclusively in extracts of *Ficus hispida* fruits, appear to induce enhancement of Bcl-2 [64–68]. Also, chlorogenic acid, gallic acid, alpinumisoflavone, (-)epicatechin, rosmarinic acid suppress ROS generation [69–73]. Gallic acid also impedes any effect originating from DNA damage [74]. Both gallic acid and chlorogenic acid can inhibit the binding of TNF-α to the death receptor TNFR, thus leading to the deactivation of extrinsic apoptosis [75]. Moreover, our study findings have demonstrated that chlorogenic acid, gallic acid, alpinumisoflavone, and betulinic acid -all four have the potential to attenuate the activity of caspase-3, a key regulator of pancreatic β-cell apoptosis. In this regard, *Ficus hispida* fruit is regarded to emerge as a prospective alternative to current anti-diabetic drugs having dual involvement in suppressing the leading enzymes involved in the development of Type 2 diabetes mellitus.

Despite the promising findings obtained through in-vitro and in-silico experiments, our study cannot completely reflect the inherent complexity of biological system. The absence of in-vivo animal models and mammalian cell-line models restrict our capability to accurately estimate pharmacokinetics and pharmacodynamics behaviour, systemic bioavailability, dose extrapolation, and efficacy of our tested plant extract in the diverse physiological environment of living organisms [76,77]. Similarly, lack of experimental toxicity studies (acute or chronic toxicity tests) hinder the establishment of safe exposure thresholds, appropriate dosing decisions, while also obscuring possible prolonged harmful consequences [78,79]. The findings obtained from in-silico based experiments only offer preliminary insights, rather than firm evidence of therapeutic efficacy, highlighting the substantial discrepancy between predictive computational modelling and real-world experimental validation [80].

## 5. Conclusion

*F. hispida* fruit extract showed promising antidiabetic activity by *in-vitro* assay. (-) Epicatechin, chlorogenic acid, alpinumisoflavone, isowighteone hydrate, rosmarinic acid, and myricetin were identified by docking study as the most effective compounds against antidiabetic targets such as α-glucosidase. In addition, network pharmacology, molecular docking, and dynamics simulation studies of gallic acid and alpinumisoflavone demonstrated potent inhibitory activity against the caspase-3 enzyme, thus counteracting the reduction of pancreatic β-cell mass. As this study is bounded by exclusive use of in-vitro and in-silico approaches, further investigation is necessary to elucidate the exact mechanism and effect of the extract and its constituents through long-term studies on animal models and cell-line based assays.

## Supporting information

S1 Table Inhibitory pattern of FhME fruit extract against α-glucosidase at different concentrations. (PDF)

S2 Table List of identified polyphenolic compounds from the methanol extract of fruits of *F. hispida* via HPLC-DAD analysis. (PDF)

S3 Table Correlation Coefficient (R²), Limit of Detection (LOD), and Limit of Quantiﬁcation (LOQ) of HPLC-DAD analysis. (PDF)

S4 Table Chemical constituents of *F. hispida* fruits adopted from Zhang et al., (2018); Cheng et al. (2021). (PDF)

S5 Table 11 selected compounds with their associated disease genes of T2DM. (PDF)

S6 Table Representing the amino acids residues that bind between protein and selected three compounds. (PDF)

S7 Table The physico-chemical properties of these selected compounds. (PDF)

S8 Table ADMET properties of three selected compounds. (PDF)

S9 Table Calculated binding free energies from post-docking MM-GBSA of selected compounds. (PDF)
