# Supplementary material for: Mechanistic insight into the antidiabetic effects of Ficus hispida fruits: Inhibition of intestinal glucose absorption and pancreatic beta-cell apoptosis
Source: PLoS One. 2025 Dec 1;20(12):e0337465. doi: 10.1371/journal.pone.0337465 (PMC12668534; doi:10.1371/journal.pone.0337465)
Supplement: S1 Table — (PDF) [file pone.0337465.s001.pdf]

Supplementary Table 1: Inhibitory pattern of FhME fruit extract against α-glucosidase at different concentrations

Concentration (mg/ml)	FhME			Acarbose		
	Mean	Standard deviation (SD)	Number of replicates (N)	Mean	Standard deviation (SD)	Number of replicates (N)
0.01	7.19	1.2	4	13.15	1.1	4
0.05	10.15	1.1	4	17.05	1.5	4
0.1	11.24	2.2	4	26.63	1.2	4
0.2	24.32	2	4	41.11	2.45	4
0.4	34.26	2.6	4	43.57	1.2	4
0.6	39.25	1.2	4	58.02	2.6	4
0.8	46.37	2.7	4	62.08	2.2	4
1	67.74	2.8	4	71.44	1.2	4
2.5	74.71	2.2	4	76.26	2.6	4
