# Supplementary material for: Mechanistic insight into the antidiabetic effects of Ficus hispida fruits: Inhibition of intestinal glucose absorption and pancreatic beta-cell apoptosis
Source: PLoS One. 2025 Dec 1;20(12):e0337465. doi: 10.1371/journal.pone.0337465 (PMC12668534; doi:10.1371/journal.pone.0337465)
Supplement: S2 Table — (PDF) [file pone.0337465.s002.pdf]

Supplementary Table 2: List of identified polyphenolic compounds from the methanol extract of fruits of *F. hispida* via HPLC-DAD analysis

**Name of Standard**	***F. hispida*** **(mg/100 g dry extract)**
Gallic acid	Nd.
3,4 dihyrdoxy benzoic acid	Nd.
Catechin hydrate	25.32±0.26
Catechol	Nd.
(-) Epicatechin	18.91±0.65
Caffeic acid	Nd.
Vanillic acid	Nd.
Syringic acid	Nd.
Rutin hydrate	3.82±0.17
p-Coumaric acid	Nd.
Trans-Ferulic acid	1.77±0.02
Rosmarinic acid	4.87±0.10
Myricetin	1.24±0.11
Quercetin	Nd.
Trans-Cinnamic acid	Nd.
Kaempferol	0.48±0.04

Nd-Not Detected
