# Supplementary material for: Mechanistic insight into the antidiabetic effects of Ficus hispida fruits: Inhibition of intestinal glucose absorption and pancreatic beta-cell apoptosis
Source: PLoS One. 2025 Dec 1;20(12):e0337465. doi: 10.1371/journal.pone.0337465 (PMC12668534; doi:10.1371/journal.pone.0337465)
Supplement: S3 Table — (PDF) [file pone.0337465.s003.pdf]

Supplementary Table 3: Correlation Coefficient (R^2), Limit of Detection (LOD), and Limit of quantification (LOQ) of HPLC-DAD analysis

Standard Compounds	*R^2	*LOD (ppm)	*LOQ (ppm)	Function
Catechin hydrate	0.9996477	0.16	0.48	f(x)=6.4134e-005*x-0.462459
(-) Epicatechin	0.9998733	0.15	0.44	f(x)=5.24198e-005*x-0.0477038
Rutin hydrate	0.9999276	0.07	0.23	f(x)=1.74671e-005*x+0.279518
Trans-Ferulic acid	0.9999365	0.09	0.26	f(x)=1.61095e-005*x-0.201679
Rosmarinic acid	0.9999681	0.16	0.49	f(x)=2.80317e-005*x+0.529651
Myricetin	0.9999029	0.10	0.30	f(x)=1.20032e-005*x+0.194481
Kaempferol	0.9996267	0.11	0.33	f(x)=5.50516e-006*x+0
