# Supplementary material for: Mechanistic insight into the antidiabetic effects of Ficus hispida fruits: Inhibition of intestinal glucose absorption and pancreatic beta-cell apoptosis
Source: PLoS One. 2025 Dec 1;20(12):e0337465. doi: 10.1371/journal.pone.0337465 (PMC12668534; doi:10.1371/journal.pone.0337465)
Supplement: S4 Table — (PDF) [file pone.0337465.s004.pdf]

Supplementary Table 4: Chemical constituents of *F. hispida* fruits adopted From (Zhang et al., 2018; Cheng et al. (2021).

**Chemical class**	**Compound**
Triterpenoids	Betulinic acid
Flavonoids	Isowigtheone hydrate
	3′-Formyl-5,7-dihydroxy-4′ methoxyisoflavone
	5,7-Dihydroxy-4′-methoxy-3′-(3-methyl-2-hydroxybuten-3-yl)isoflavone
	Alpinumisoflavone
Coumarins	7-Hydroxy-6-[2-(R)-hydroxy-3-methyl-but- 3-enyl]coumarin
	7-Hydroxycoumarin
	(–)-Marmesin
Phenylpropionic acids	Chlorogenic acid
	Chlorogenic acid methyl ester
	Chlorogenine glycoside
Benzoic acid derivatives	Gallic acid
	Protocatechuic acid
Alkaloids	Murrayaculatine
Steroids	Sitosterol 3-O-β-D-glucopyranoside
Glycosides	2-(4-Hydroxy-3-methoxyphenyl)ethyl β-D-glucopyranoside
	(6S,9R)-Roseoside
	Benzyl β-D-glucopyranoside
Psoralens	Psoralen
