# Supplementary material for: Mechanistic insight into the antidiabetic effects of Ficus hispida fruits: Inhibition of intestinal glucose absorption and pancreatic beta-cell apoptosis
Source: PLoS One. 2025 Dec 1;20(12):e0337465. doi: 10.1371/journal.pone.0337465 (PMC12668534; doi:10.1371/journal.pone.0337465)
Supplement: S5 Table — (PDF) [file pone.0337465.s005.pdf]

Supplementary Table 5: 11 selected compounds with their associated disease genes of T2DM

Compounds	Disease genes
Betulinic acid	LMNB1
Betulinic acid	CASP3
Betulinic acid	SP1
Betulinic acid	TOP2A
Betulinic acid	NOS3
Betulinic acid	AKT1
Betulinic acid	CYCS
Betulinic acid	BIRC5
Betulinic acid	PNLIP
Betulinic acid	TOP1
Myricetin	SLC2A2
Myricetin	PIK3CG
Myricetin	TOP2A
Myricetin	AKT1
Myricetin	BMP2
Myricetin	SIRT1
Myricetin	CYP1B1
Myricetin	ALOX5
Myricetin	PIM1
Myricetin	AMY2A
Gallic acid	MMP2
Gallic acid	TYR
Gallic acid	ABCB1
Gallic acid	ATM
Gallic acid	CASP3
Gallic acid	JUN
Gallic acid	GATA3

Gallic acid	EIF2AK3
Gallic acid	SERPINE1
Kaempferol	CDK1
Kaempferol	AHR
Kaempferol	UGT1A8
Kaempferol	UGT1A7
Kaempferol	NR1I2
Kaempferol	CYP1B1
Kaempferol	UGT1A9
Kaempferol	RPS6KA3
Protocatechuic acid	DPYD
Protocatechuic acid	AHCY
Protocatechuic acid	NR5A1
Protocatechuic acid	MPO
Protocatechuic acid	COMT
Protocatechuic acid	NR0B1
Protocatechuic acid	ADSL
Protocatechuic acid	DHODH
Chlorogenic acid	UGT1A8
Chlorogenic acid	UGT1A7
Chlorogenic acid	TYR
Chlorogenic acid	HMGB1
Chlorogenic acid	CASP3
Chlorogenic acid	UGT1A10
Chlorogenic acid	MAPK8
Chlorogenic acid	DNMT1
Rosmarinic acid	CCR3
Rosmarinic acid	PARG
Rosmarinic acid	PROCR
Rosmarinic acid	FOS

Rosmarinic acid	IKBKB
Rosmarinic acid	LCK
Rosmarinic acid	IL2
Trans-Ferulic acid	CYP1A1
Trans-Ferulic acid	UGT1A8
Trans-Ferulic acid	UGT1A7
Trans-Ferulic acid	G6PD
Trans-Ferulic acid	TYR
Trans-Ferulic acid	CYCS
Trans-Ferulic acid	DHFR
Rutin hydrate	GSR
Rutin hydrate	EGFR
Rutin hydrate	AKR1C3
Rutin hydrate	ALDH2
Rutin hydrate	SREBF1
Rutin hydrate	HSPA4
Rutin hydrate	P4HB
Alpinumisoflavone	STAT5A
Alpinumisoflavone	STAT3
Alpinumisoflavone	STAT6
Alpinumisoflavone	CASP3
Alpinumisoflavone	CASP9
Alpinumisoflavone	PARP1
7-Hydroxycoumarin	UGT2B15
7-Hydroxycoumarin	CYP2A6
7-Hydroxycoumarin	MPO
7-Hydroxycoumarin	CCND1
7-Hydroxycoumarin	UGT1A9
