# Supplementary material for: Mechanistic insight into the antidiabetic effects of Ficus hispida fruits: Inhibition of intestinal glucose absorption and pancreatic beta-cell apoptosis
Source: PLoS One. 2025 Dec 1;20(12):e0337465. doi: 10.1371/journal.pone.0337465 (PMC12668534; doi:10.1371/journal.pone.0337465)
Supplement: S6 Table — (PDF) [file pone.0337465.s006.pdf]

Supplementary Table 6: Representing the amino acids residues that bind between protein and selected three compounds.

**Compound Name**	**CID**	**H-bond**	**Polar bond**	**Hydrophobic bond**
Gallic acid	CID: 370	THR 167, LEU 168, LYS 259	THR 166, THR 255	LEU 168, CYS 170
Alpinumisoflavone	CID: 5490139	SER 63	THR 62, THR 63, SER 65, HIE 121, THR 166, SER 209	LEU 168, TYR 204, PHE 256
Chlorogenic Acid	CID: 1794427	SER 63, SER 65, ARG 207	THR 62, SER 63, SER 65, HIE 121, THR 166, SER 209	LEU 168, TYR 204, PHE 256
