# Supplementary material for: Mechanistic insight into the antidiabetic effects of Ficus hispida fruits: Inhibition of intestinal glucose absorption and pancreatic beta-cell apoptosis
Source: PLoS One. 2025 Dec 1;20(12):e0337465. doi: 10.1371/journal.pone.0337465 (PMC12668534; doi:10.1371/journal.pone.0337465)
Supplement: S7 Table — (PDF) [file pone.0337465.s007.pdf]

Supplementary Table 7: The physico-chemical properties of these selected compounds.

**Properties**	**Selected compounds**		
	**Chlorogenic acid**	**Alpinumisoflavone**	**Gallic acid**
**Molecular weight**	354.31	336.34	170.12
**H-bond donors**	6	2	4
**H-bond acceptors**	9	5	5
**Rotatable bonds**	5	1	1
**Consensus Log $P_{o/w}$**	-0.38	3.18	0.21
**TPSA**	164.75	79.90	97.99
**Lipinski violation**	Yes;1 violation	Yes; 0 violation	Yes; 0 violation
