# Supplementary material for: Mechanistic insight into the antidiabetic effects of Ficus hispida fruits: Inhibition of intestinal glucose absorption and pancreatic beta-cell apoptosis
Source: PLoS One. 2025 Dec 1;20(12):e0337465. doi: 10.1371/journal.pone.0337465 (PMC12668534; doi:10.1371/journal.pone.0337465)
Supplement: S8 Table — (PDF) [file pone.0337465.s008.pdf]

Supplementary Table 8: ADMET properties of three selected compounds.

ADMET	Properties	Selected compounds		
		**Chlorogenic acid**	**Alpinumisoflavone**	**Gallic acid**
**Bioavailability**	**Bioavailability score**	0.11	0.55	0.56
**Absorption**	**Water solubility (log mol/L)**	-2.449	-3.603	-0.723
	**Human intestinal absorption (% absorbed)**	36.38	94.521	50.311
	**GI absorption**	Low	High	High
	**Caco2 permeability (log Papp in 10-6 cm/s)**	-0.84	1.163	-0.467
	**Log K_p (skin permeation) (cm/s)**	-8.76	-5.55	-6.84
**Distribution**	**BBB permeability (log BB)**	-1.407	0.074	-0.93
	**CNS permeability (log PS)**	-3.856	-1.882	-2.816
	**VDss (human) (log L/kg)**	0.581	0.234	-1.078
**Metabolism**	**CYP1A2 inhibitior**	No	Yes	No
	**CYP2C19 inhibitior**	No	Yes	No
	**CYP2C9 inhibitior**	No	Yes	No
	**CYP2D6 inhibitior**	No	No	No
	**CYP3A4 inhibitior**	No	Yes	Yes
**Excretion**	**Total Clearance (log ml/min/kg)**	0.307	0.202	0.55
	**Renal OCT2 substrate**	No	No	No

**Toxicity**	**AMES toxicity**	No	No	No
	**Max. tolerated dose (human)**	-0.13	0.014	1.404
	**hERG I inhibitor**	No	No	No
	**hERG II inhibitor**	No	Yes	No
	**Oral Rat Acute Toxicity (LD50) (mol/kg)**	1.973	2.476	1.872
	**Oral Rat Chronic Toxicity (LOAEL) (log mg/kg_bw/day)**	2.982	1.588	1.499
	**Hepatotoxicity**	No	Yes	No
