# Supplementary material for: Mechanistic insight into the antidiabetic effects of Ficus hispida fruits: Inhibition of intestinal glucose absorption and pancreatic beta-cell apoptosis
Source: PLoS One. 2025 Dec 1;20(12):e0337465. doi: 10.1371/journal.pone.0337465 (PMC12668534; doi:10.1371/journal.pone.0337465)
Supplement: S9 Table — (PDF) [file pone.0337465.s009.pdf]

Supplementary Table 9: Calculated binding free energies from post-docking MM-GBSA of selected compounds

MM-GBSA calculations	Selected compounds		
	Chlorogenic acid	Alpinumisoflavone	Gallic acid
dG Bind	-27.43	-30.96	-17.58
dG Bind Coulomb	-26.9	-20.43	2.56
dG Bind Covalent	5.49	0.46	2.99
dG Bind Hbond	-3.65	-1.47	-2.42
dG Bind Lipo	-11.35	-6.55	-4.81
dG Bind Packing	-2.61	-3.09	0
dG Bind SelfCont	0	0	0
dG Bind Solv GB	32.37	22.98	-2.9
dG Bind vdW	-20.79	-22.86	-13.01
